# Metabolite Profiling of Lavender (*Lavandula pedunculata* subsp. *cariensis*) Essential Oil and Investigation of Its Potential Antioxidant and Enzyme-Inhibitory Effects

**DOI:** 10.3390/ph19060966

**Published:** 2026-06-22

**Authors:** Hasan Karageçili, Eda Mehtap Özden, Muzaffer Mutlu, Zeynebe Bingöl, Hülya Akıncıoğlu, Ekrem Köksal, Ahmet Ceyhan Gören, İlhami Gülçin

**Affiliations:** 1 Department of Nursing, Faculty of Health Sciences, Siirt University, Siirt 56100, Türkiye; 2 Department of Chemistry, Faculty of Science, Ataturk University, Erzurum 25240, Türkiye; edamehtap3@gmail.com; 3 Vocational School of Applied Sciences, Gelişim University, Istanbul 34315, Türkiye; muzaffermutlu@hotmail.com; 4 Department of Medical Services and Techniques, Tokat Vocational School of Health Services, Gaziosmanpasa University, Tokat 60250, Türkiye; zeynep.bingol196@gmail.com; 5 Department of Biochemistry, Faculty of Pharmacy, Ağrı İbrahim Çeçen University, Agri 04100, Türkiye; hakincioglu@agri.edu.tr; 6 Department of Chemistry, Faculty of Science and Arts, Erzincan Binali Yildirim University, Erzincan 24100, Türkiye; koksalekrem@gmail.com; 7 Department Chemistry, Faculty of Sciences, Gebze Technical University, Kocaeli 41400, Türkiye; acgoren@gtu.edu.tr; 8 Rectorate of Agri Ibrahim Çeçen University, Agri 04100, Türkiye

**Keywords:** *Lavandula cariensis*, *Lavandula pedunculata*, essential oil, antioxidant activity, GC/MS, enzyme inhibition

## Abstract

**Background/Objectives:** *Lavandula cariensis* species is cultivated uncommonly in the western region of Turkey. The colloquial appellations avayianos, karabasi, and myra are used to refer to the *L. cariensis* plant. The essential oil of *L. cariensis* was studied for its potential antiglaucoma, antioxidant, antidiabetic, and acetylcholinesterase inhibitory effects. **Methods:** The inhibitory effect of the essential oil of *L. cariensis* on the acetylcholinesterase (AChE), carbonic anhydrase II (CA II), and α-amylase enzymes was determined. Therefore, chemical profiles of *L. cariensis’* essential oil were identified using Gas Chromatography Mass Spectrometry (GC-MS) and as Chromatography with Flame Ionization Detection (GC-FID) analyses. **Results:** Camphor (39.73%), fenchone (19.49%), exobornyl acetate (6.81%), camphene (5.49%), and eucalyptol (5.49%) were the most abundant compounds in *L. cariensis* essential oil. Radical scavenging effect of the essential oil of *L. cariensis* was examined using 1,1-diphenyl-2-picrylhydrazyl (DPPH) (IC_50_: 231.0 ± 0.094 μg/mL) and 2,2′-azino-bis(3-ethylbenzthiazoline-6-sulfonic acid) (ABTS) (IC_50_: 7.45 ± 0.013 μg/mL) radicals. Also, the ferric ions (Fe^3+^), cupric ions (Cu^2+^), and Fe^3+^-2,4,6-tri(2-pyridyl)-S-triazine (TPTZ) complex reducing capabilities were studied. Additionally, essential oil of *L. cariensis* indicated a comparable level of inhibition towards hCA II (IC_50_: 276.42 μg/mL), AChE (IC_50_: 14.22 μg/mL), and α-amylase (IC_50_: 475.63 μg/mL) enzymes. **Conclusions:** The evaluation of the antioxidant capabilities and enzyme inhibition profiling of the essential oil of *L. cariensis* will be made possible by this comprehensive study, which serves as a springboard for further research. The essential oil of *L. cariensis* demonstrated enzyme-inhibitory activities against target enzymes associated with Alzheimer’s disease, diabetes, and glaucoma. Also, this study’s in vitro inhibition suggests promising prospects.

## 1. Introduction

As a reservoir of bioactive substances applied to alleviate many human ailments, medicinal plants are of tremendous concern. Essential oils are used in the aromatic and pharmaceutical sectors as secondary metabolites. Consequently, their use in pharmaceutical, cosmetic, and food applications has increased considerably over the past decade. To cure human diseases, many studies have recommended using essential oils rather than manufactured pharmaceuticals [1]. Essential oils are typically lipophilic extracts made up of several monoterpenoids and sesquiterpenoids, which are mostly extracted by steam distillation from flowers, herbs, and spices. The proportions of these compounds vary by species. Throughout history, they have been extensively utilised as analgesics, anti-inflammatories, bacteriostatics, diuretics, expectorants, and fungicides in addition to aromatherapy, flavouring, and fragrances. Thus, the chemical, cosmetic, food, fragrance, and pharmaceutical sectors are now researching and using essential oils [2]. Most aromatic and medicinal plants in the world are found in the Lamiaceae (Labiatae) family, which has 7886 species in about 245 genera. There are 46 genera and 782 taxa that belong to this family in Turkey [3]. Particularly among the most important families for producing essential oils is probably the Lamiaceae family, especially members of the *Lavandula* genus, including *L. angustifolia* and *L. stoechas* [4]. Because of their antioxidant action, lavender species show promise for a variety of biological uses. The development of medications may make it easier to identify and characterise various bioactive substances. Through in vitro and in vivo research, the collaborative benefits of lavender’s bioactive ingredients with other compounds show promise for treating illnesses associated with compromised immune systems and oxidative stress. The main components of lavender vary in relative quantities across species, comprising volatile oils such as linalool, limonene, perillyl alcohol, linalyl acetate, *cis*-smine, terpene, coumarin, tannin, caffeic acid, and camphor [5]. The colloquial appellations avayianos, karabasi, and myra are used to refer to *L. cariensis* [6]. Traditionally, tea prepared from the dried flowers and leaves of *L. pedunculata* subsp. cariensis has been prescribed as a mucolytic, tranquillizer, and remedy for stomachaches, tremors, bronchitis, embolism, and hypertension [3]. High quantities of linalool and linalyl acetate, together with modest amounts of lavandulyl acetate, terpinen-4-ol, and lavandulol, are characteristics of lavender essential oil. Camphor and 1,8-cineole concentrations are frequently extremely low to moderate [7]. *L. stoechas* essential oils and extracts have been shown to have antidiabetic, antioxidant, antiamnesic, and antibacterial properties [8]. *L. stoechas* has been widely utilised in Morocco to treat nephrotic syndrome, rheumatoid arthritis, and as an antispasmodic to reduce inflammation and pain. It is also used as an infusion or decoction for its antidiabetic properties [9]. The anti-inflammatory, antifungal, antibacterial, antioxidant, sedative, and neuroprotective properties of *L. stoechas* essential oils are well-established. They have been utilised to treat wounds, burns, skin injuries, Alzheimer’s disease (AD), food, cosmetics, perfumery, and medical [10].

Several *Lavandula* species have been extensively investigated for their biological properties, particularly their antioxidants, antidiabetic, and neuroprotective activities. Essential oils from *L. stoechas* have demonstrated considerable antioxidant activity in both DPPH and ABTS radical scavenging assays, together with antidiabetic effects associated with the modulation of carbohydrate-metabolising enzymes and improvement of oxidative stress parameters in diabetic models [11]. Both methods are among the most widely used antioxidant assays [12]. Likewise, essential oils of *L. angustifolia* have been reported to exhibit significant free radical scavenging activity and protective effects against oxidative damage, largely attributed to their monoterpene-rich composition [13]. In addition, extracts and essential oils obtained from several Lavandula species, including *L. stoechas* and *L. dentata*, have shown acetylcholinesterase (AChE) inhibitory effect, suggesting potential relevance in the management of neurodegenerative disorders associated with cholinergic dysfunction [14]. These findings indicate that members of the genus Lavandula represent promising sources of natural compounds with multifunctional biological properties. However, despite the increasing number of studies on other lavender species, information regarding the antioxidant and enzyme-inhibitory activities of *L. cariensis* essential oil remains scarce. Therefore, further investigation of its chemical composition and biological potential is warranted.

Common edible plant meals include phenolic chemicals, which are physiologically active secondary metabolites. Via donating hydrogen, scavenging free radicals, functioning as chelators, and quenching reactive oxygen species (ROS), phenolic compounds have antioxidant action [15,16]. Antioxidants have grown significantly during the last ten years to avoid damage caused by oxidative stress. Furthermore, plants are an excellent source of natural antioxidants and a safe substitute for synthetic compounds such as butylated hydroxyanisole (BHA) and butylated hydroxytoluene (BHT), which are frequently utilised as preservatives in consumer products and food and have been connected to doubtful carcinogenic and toxic properties [17]. Through neutralising harmful free radical reactions and/or quenching reactive metals that produce ROS, flavonoids and polyphenols are strong dietary scavengers of oxidant precursors that protect against illnesses caused by oxidative stress. Additionally, these scavengers raise antioxidant capacity, improve the activity of antioxidant enzymes, repair oxidised membranes, and lower peroxide concentrations. Regular nutritious food is very appealing as an alternative preventative therapy remedy towards degenerative illnesses since fruits and vegetables contain a variety of flavonoids and polyphenols [18]. Medicinal herbs, which have been the subject of several studies to date, constitute the most prominent reservoirs of natural antioxidants. Phenols are found in significant quantities in therapeutic plants. Fruits, vegetables, and grains are the main dietary sources of natural antioxidants for the typical human. Secondary metabolites of plants called phenolic compounds offer protection from degenerative diseases such as rheumatoid arthritis, diabetes, heart disease, cancer, cataracts, and high cholesterol [19]. Flavonoids are antioxidant phenols that scavenge free radicals from tissues as well as organs in diets rich in plants. The vascular endothelium functions better as a consequence of dietary plant ingredients, lowering the risk of high blood pressure, diabetes, AD, and other cardiovascular conditions [20].

Alzheimer’s disease (AD) is a rapidly progressing neurological condition that causes behavioural changes, memory loss, cognitive decline, and language impairments. Acetate (CH_3_COO^−^) and choline (Ch) are produced when acetylcholinesterase (AChE) breaks down acetylcholine (ACh) [21]. The cholinergic theory states that AD patients have reduced brain levels of the neurotransmitter ACh, which results in a lack of memory [22]. AD is categorised as a progressive neurological condition with incremental growth and impairment in function due to the death of neurons in the central nervous system. The pathophysiology of AD is known to be significantly influenced by acute intake of the acetylcholine neurotransmitter [23]. The most often recommended drugs for AD are acetylcholinesterase inhibitors, which include galantamine, rivastigmine, donepezil, and tacrine. They enhance cognitive function and offer therapeutic comfort. By increasing the supply of acetylcholine at cholinergic synapses, they also enhance cholinergic neurotransmission. However, some of these medications’ drawbacks include hepatotoxicity, low absorption, severe cholinergic side effects, and limited efficacy [24]. Tacrine, galantamine, donepezil, and rivastigmine are among the most prevalent cholinesterase inhibitors. Medical therapy withdrawal is a common outcome of these pharmaceutical inhibitors’ side effects. Some of the most severe adverse effects include constipation, hepatotoxicity, nausea, vomiting, and diarrhoea [25,26].

Diabetes continues to be a global issue because of the large number of fatalities it results in, despite advancements in antihyperglycemic and antidiabetic drugs. Although there are new medicinal substances like insulin and oral hypoglycaemic medications, regular use of them is associated with several negative consequences [9]. Although plants and compounds produced from them are utilised for the treatment of AD, they also affect the biochemical pathways that lead to diabetes-related issues. In the past, low blood sugar levels and diabetes were treated using medications derived from plants, such as metformin [27].T2DM, on the contrary, is a physiological problem brought on by elevated levels of glucose in the blood and is a contributing factor in several health issues, including gangrene, cardiovascular disease, neuropathy, nephropathy, and high blood pressure [28]. Similarly, the enzymes α-amylase and α-glycosidase, which are released by small intestine cells, hydrolyse oligosaccharide and polysaccharide molecules into monosaccharide units that include glucose and fructose. Digestive enzyme inhibitors have been demonstrated to treat T2DM and postprandial hyperglycaemia by decreasing the absorption of dietary carbs. Consequently, they effectively reduce postprandial polysaccharide units in T2DM, especially glucose [29]. Digestive enzyme inhibitors (DEIs), which are available in natural products, can thus be used to alleviate T2DM and hyperglycaemia. DEIs lower blood sugar levels and have an antidiabetic impact since they reduce intestinal glucose absorption. Consequently, oligosaccharides and DEIs fight for binding to the active site of the enzyme. Acarbose is a typical inhibitor for this kind of inhibition [30].

Glaucoma is a multifactorial visual illness that is primarily caused by high intraocular pressure (IOP), which can lead to blindness. hCA inhibitors, such as acetazolamide and dorzolamide, are effective in lowering IOP after topical application; however, these therapies have a number of side effects that call for the creation of innovative treatment methods [28]. Actually, CA isoforms offer a broad therapeutic approach, and the development of isoenzyme-specific inhibitors is a great way to create a drug with minimal side effects [31]. Acetazolamide, methazolamide, ethoxzolamide, dichlorphenamide, dorzolamide, brinzolamide, and other powerful CA inhibitors have been utilised in clinical settings for decades as diuretics, antiglaucoma medications, and antiepileptics [32]. The CA isoforms present in several tissues outside the eye are inhibited by these inhibitors, which is why they are known to have unwanted adverse effects, including weariness, paraesthesia, or increased urination [33]. To avoid these undesirable adverse effects, it is best to utilise inhibitors with topical action that comes from nature. Therefore, we bring the use of *L. cariensis* essential oil in treatment to the attention of glaucoma researchers. In this study, we evaluated *L. cariensis* essential oil’s chemical composition, antioxidants, anti-AD, antiglaucoma, and antidiabetic abilities. We also calculated the essential oil of *L. cariensis* using GS-MS/FID.

*L. cariensis *(syn. *L. pendunculata* subsp.* cariensis*) is a geographically restricted and relatively understudied species of the genus *Lavandula*, naturally distributed in western Türkiye and certain Aegean regions. Unlike the extensively investigated species such as *L. angustifolia* and *L. stoechas*, limited information is available regarding its phytochemical composition and biological properties. The species is characterised by a distinctive essential oil profile rich in oxygenated monoterpenes, particularly camphor and fenchone, which are known to possess various biological activities. Therefore, the investigation of *L. cariensis* is important not only for expanding the phytochemical knowledge of the genus but also for identifying novel natural sources of antioxidant and enzyme-inhibitory compounds with potential pharmaceutical, nutraceutical, and industrial applications.

Also, to the best of our knowledge, this study represents the first comprehensive investigation of the chemical composition, antioxidant potential, and multi-enzyme-inhibitory activities of *L. cariensis* essential oil. The work provides novel insights by integrating GC-MS/GC-FID characterisation with evaluations of AChE, hCA II, and α-amylase inhibitory effects, thereby expanding the pharmacological knowledge of this poorly studied endemic lavender species. Compositional profiling, antioxidant assays, α-amylase, and AChE inhibition have been widely reported for *L. angustifolia*, *L. stoechas*, *L. pinnata*, and other lavender taxa. The unique chemotype of *L. cariensis*, distinguished by high camphor (39.73%) and fenchone (19.49%) contents, further supports its phytochemical distinctiveness.

The aim of this study was to characterise the chemical composition of *L. cariensis* essential oil using GC-MS and GC-FID analyses and to evaluate its antioxidant and enzyme-inhibitory properties. In this context, the radical-scavenging, metal-reducing, anticholinesterase, antidiabetic, and antiglaucoma abilities of the essential oil were investigated through in vitro bioanalytical assays. Additionally, the inhibitory effects of the essential oil against AChE, α-amylase, and hCA II enzymes were determined to explore its potential as a natural source of bioactive compounds with multifunctional biological activities.

## 2. Results

### 2.1. Chemical Composition of Lavandula Cariensis Oil

**Figure 1:**
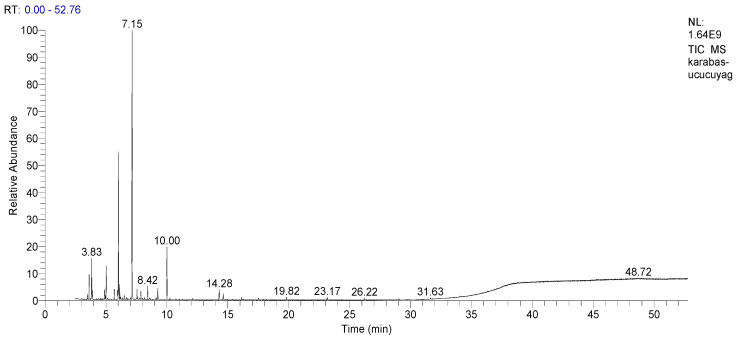
GC–MS Chromatogram of the essential oil sample obtained from *L. cariensis.*

GC-MS was used to accurately and qualitatively analyse the constituents of various aromatic substances and essential oils. Table 1 displays the relative details of *L. cariensis* essential oil’s aromatic ingredients. The present research identified five volatile compounds in *L. cariensis* essential oil specimens. Camphor (39.73%), fenchone (19.49%), exobornyl acetate (6.81%), camphene (5.49%), and eucalyptol (5.49%) were the most prevalent substances in *L. cariensis* essential oil (Table 1 and Figure 1). In the studies conducted, only 96.44% of the total components were identified. The remaining 3.56% of the essential oil consisted of trace constituents present at very low concentrations and/or compounds that could not be reliably identified due to insufficient spectral matching or peak overlap during GC–MS analysis.

### 2.2. Reducing the Ability of L. cariensis Essential Oil

**Figure 2:**
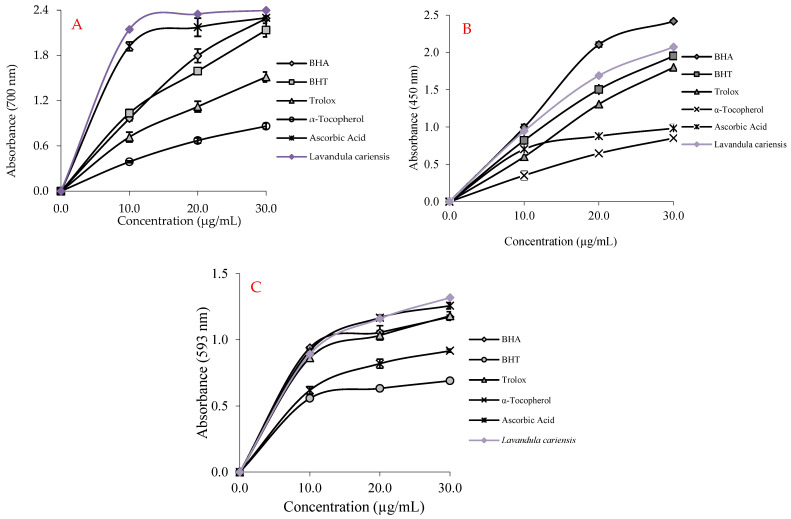
Ferrous ions (Fe^3+^) (**A**), Cupric ions (Cu^2+^) (**B**), and Fe^3+^-TPTZ complex (**C**) reducing ability of *L. cariensis* essential oil and standards.

*L. cariensis* essential oil exhibited a powerful reducing capability in the Fe[Fe(CN^−^)_6_]_3_, Fe^3+^-TPTZ, and Cu^2+^ reduction experiments. A Fe^3+^–Fe^2+^ transformation assay was initially conducted to ascertain the reducing power of *L. cariensis* essential oil (Figure 2A and Table 2). At a concentration of 30 µg/mL, *L. cariensis* essential oil and standards showed the potential to reduce Fe^3+^ (*p* < 0.01) as follows: *L. cariensis* essential oil (2.397 ± 0.093, r^2^ = 0.9600) ≥ Ascorbic acid (2.298 ± 0.086, r^2^ = 0.9659) ≥ BHA (2.292 ± 0.012, r^2^ = 0.9993) ≥ BHT (2.136 ± 0.090, r^2^ = 0.9957) > Trolox (1.514 ± 0.066, r^2^ = 0.9963) > α-Tocopherol (0.862 ± 0.038, r^2^ = 0.9996). A high absorbance value in the measurement of the samples is the result of high reducing activity (Figure 2A).

Figure 2B, C, and Table 2 give an overview of the research into *L. cariensis* essential oil’s Fe^3+^-TPTZ and Cu^2+^-reducing features in addition to its Fe^3+^-reducing action. At the evaluation quantities, *L. cariensis* essential oil had higher absorbance levels. The following is the subsequent order of the standards, *L. cariensis* essential oil to reduce Cu^2+^ ions at a quantity of 30 μg/mL: (Figure 2B): BHA (2.418±0.018, r^2^ = 0.9887) > *L. cariensis* essential oil (2.073 ± 0.105, r^2^ = 0.9995) > BHT (1.953 ± 0.045, r^2^ = 0.9998) > Trolox (1.800 ± 0.096, r^2^ = 0.9974) > Ascorbic acid (0.983 ± 0.048, r^2^ = 0.9822) > α-Tocopherol (0.851 ± 0.046, r^2^ = 0.9994).

The *L. cariensis* essential oil showed an effective reducing capability in this reduction assay (Table 2 and Figure 2C). The ability to reduce FRAP was decreased in the subsequent order in test items consist of *L. cariensis* essential oil and standards: *L. cariensis* essential oil (1.318 ± 0.021, r^2^ = 0.9873) > Ascorbic acid (1.257 ± 0.024, r^2^ = 0.9869) > Trolox (1.180 ± 0.032, r^2^ = 0.9732) ≥ BHA (1.172 ± 0.014, r^2^ = 0.9605) > α-Tocopherol (0.918 ± 0.011, r^2^ = 0.9904) > BHT (0.690 ± 0.008, r^2^ = 0.9645).

### 2.3. Radicals Scavenging Effect of L. cariensis Essential Oil

**Figure 3:**
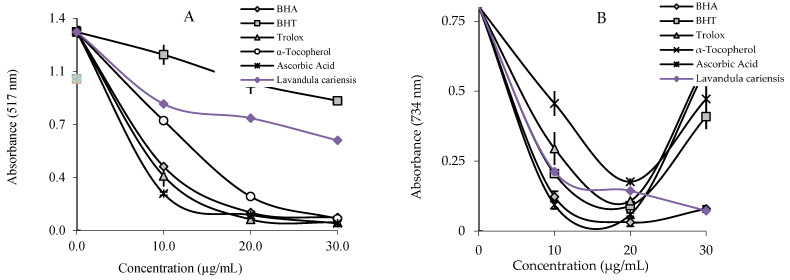
Effects of *L. cariensis* essential oil and standards on the scavenging of DPPH^•^ (**A**) and ABTS^•+^ (**B**).

The IC_50_ values for DPPH scavenging of *L. cariensis* essential oil and conventional radical scavengers are as follows: 5.82 µg/mL for Ascorbic acid (r^2^ = 0.5211) < 6.03 µg/mL for Trolox (r^2^ = 0.5964) < 6.86 µg/mL for BHA (r^2^ = 0.9762) < 7.70 µg/mL for α-Tocopherol (r^2^ = 0.5221) < 49.50 µg/mL (r^2^ = 0.9155) for BHT < 231.0 µg/mL for *L. cariensis* essential oil (r^2^ = 0.8669, see Table 3 and Figure 3A).

As given in Figure 3B, *L. cariensis* essential oil had a concentration-dependent capacity to powerfully scavenge ABTS radicals (10–30 µg/mL, *p* < 0.001). *L. cariensis* essential oil’s IC_50_ value was evaluated to be 7.45 µg/mL (r^2^ = 0.882) in the ABTS^·+^ removing experiment (Table 3). The following was recorded while IC_50_ values for reference compounds were evaluated: 6.36 µg/mL for BHA (r^2^ = 0.945) < 11.75 µg/mL for Ascorbic acid (r^2^ = 0.9082) < 12.60 µg/mL for BHT (r^2^ = 0.8668) < 16.50 µg/mL for Trolox (r^2^ = 0.9926) < 18.73 µg/mL for α-Tocopherol (r^2^ = 0.9082, Figure 3B).

### 2.4. Inhibition of Enzymes by L. cariensis Essential Oil

**Figure 4:**
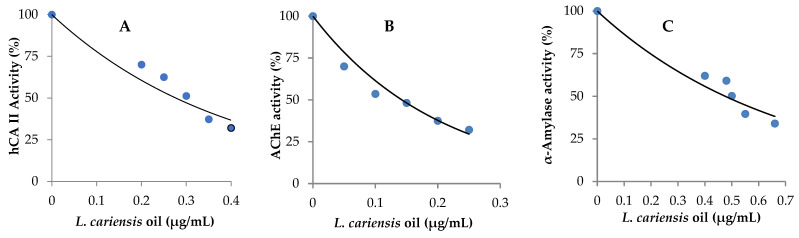
Half maximal inhibition concentration (IC_50_) graphs of *L. cariensis* essential oil towards (**A**) human carbonic anhydrase II (hCA II), (**B**) acetylcholinesterase (AChE), and (**C**) α-amylase.

Table 4 and Figure 4 consist of the results of the inhibition for applied enzymes. *L. cariensis* essential oil inhibited AChE with an IC_50_ value of 14.22 µg/mL (r^2^ = 0.9552). In the comparison investigation, the tacrine standard inhibitor inhibited AChE with an IC_50_ value of 8.82 µg/mL (r^2^ = 0.9836).

*L. cariensis* essential oil had a mildly inhibiting effect on α-amylase with an IC_50_ value of 475.63 µg/mL (r^2^ = 0.9002), as given in Table 4. With an IC_50_ value of 7.54 µg/mL (r^2^ = 0.9074), the acarbose standard inhibitor was used to inhibit α-amylase. It was found that *L. cariensis* essential oil had an IC_50_ of 276.42 µg/mL (r^2^ = 0.9055) against the dominant and cytosolic hCA II isoenzyme (Table 4). The primary and cytosolic hCA II isoforms were suppressed by the therapeutic CA isoenzyme inhibitor acetazolamide (AZA) with an IC_50_ value of 9.96 µg/mL (r^2^ = 0.9930).

## 3. Discussion

Although plant-derived polyphenols constitute an important group of secondary metabolites that function as essential antioxidants and free radical scavengers, thus contributing to various biological processes associated with improved physiological health, many non-phenolic compounds are known to play a significant role in antioxidant activity. While essential oils have been reported less frequently in this area compared to plant-derived phenolic compounds, their importance in this field has increasingly been recognized [34]. Secondary metabolites exhibiting strong antioxidant properties can be classified as flavonoids, simple phenolics, Vitamin A, ascorbic acid, and essential oils such as thymol, carvacrol, and linalool. These secondary metabolites are widely known for their impact on LDL cholesterol, DNA, and lipid oxidation, as well as their ability to suppress α-glucosidase and tyrosinase activities [35]. Herbal remedies are often used as a complement or substitute for major treatments. Because of growing curiosity in natural herbal remedies, the investigators are very interested in creating new drugs derived from the biological properties of various plants employed in traditional medical practices to cure ailments. Numerous phytochemical ingredients that have pharmacological characteristics such as anticarcinogenic, antioxidant, antibacterial, antiviral, and antimutagenic qualities are responsible for their therapeutic advantages [36]. Grains, vegetables, fruits, and medicinal and aromatic plants are extremely essential as they contain healthy antioxidants. Oxidation in biological processes plays a role in multiple illnesses, such as cancer and cardiovascular disease. Thus, consistent consumption of certain foods lowers the risk of developing these and other illnesses. Oxidative processes can be slowed down by antioxidant molecules, including those found in plants [37,38,39].

Secondary metabolites are very important for the plant kingdom through their numerous hydroxyl groups and unsaturated conjugated structures. Plant secondary metabolites are bioactive substances produced as components of the plant’s adaptation to stressful conditions, vital activities such as attraction, protection against danger, and comfort. Although they are not directly involved in the basic survival processes of reproduction and development, they are essential for the plant’s connection to its natural world [40]. While not showing as high activity as polyphenols found in leaves and flowers, essential oils can act synergistically on some antioxidant activity mechanisms. Due to their high molecular composition, they can function synergistically as reducing agents, ROS and singlet oxygen scavengers, hydrogen atom donors, and more [41]. Plant material extraction and evaluation play a significant role in the development, modernization, and quality control of herbal medicines. The aim of this study was to identify the phytochemical components of the essential oil of *L. cariensis* and to evaluate the biological activity of the essential oil.

The biological activities observed for *L. cariensis* essential oil may be associated, at least in part, with its major constituents, particularly camphor and fenchone, which together accounted for approximately 59% of the total essential oil composition. Previous studies have demonstrated that oxygenated monoterpenes, including camphor-containing essential oils, may contribute to antioxidant and enzyme-modulating activities through radical-scavenging mechanisms, redox interactions, and potential binding to enzyme active sites [14,42]. However, essential oils are highly complex mixtures, and their biological activities are generally attributed to synergistic or additive interactions among major and minor constituents rather than to a single compound alone [43].

Since natural antioxidants are safer than synthetic ones, they are the only substitute. Thus, several sources of antioxidants found in nature were identified. Numerous methods have been developed and are currently in use to assess the potential of natural antioxidants [44]. Every antioxidant assay uses a different mode of action to assess antioxidant activity. For instance, the DPPH assay assesses both hydrogen-atom and electron transfer, while the FRAP test primarily assesses the antioxidant’s ability to shift a single electron [45]. To ascertain the *L. cariensis* essential oil’s antioxidant potential, several antioxidant tests were used. Reducing the potential of plant-derived essential oils facilitates their physiological functions. Due to their potent reducing power, these essential oils reduce oxidative stress and neutralise ROS [46]. The ferric ion (Fe^3+^) reduction method can be used to precisely evaluate the reduction potential of oils or plant-based extracts. While *L. cariensis* essential oil is applied to solutions comprising Fe^3+^ ions, the blue-coloured Fe_4_[Fe(CN^−^)_6_]_3_ that results may absorb light at a wavelength of 700 nm under experimental conditions [47,48,49]. Apart from the formation of this chromophore structure, the sample mixture’s colour indicates the ability of the plant extracts to decrease in a variety of colour spectra ranging from yellow to green [50,51]. *L. cariensis* essential oil exhibited a somewhat effective reducing capacity in the reduction studies for Fe_4_[Fe(CN^−^)_6_]_3_, Fe^3+^-TPTZ, and Cu^2+^ [50,51]. The results demonstrate that *L. cariensis* essential oil’s e-donor characteristic successfully mitigates the detrimental impacts of ROS and free radicals. This chemical compound’s reductive potency was lower than that of BHT, Trolox, and BHA, but it was like that of ascorbic acid and tocopherol. Another reduction assay we used for plant extracts and oils is the CUPRAC approach, which is inexpensive, selective, dependable, and fast, and remains unaffected by hydrophobicity and chemical constituents [51]. The last reduction test used in this inquiry was the FRAP reduction procedure. Like earlier investigations, significant absorbance data show that the chemical compound has a high reduction potential. The FRAP method should be applied in acidic settings to maintain the solubility of Fe^3+^ ions.

It is well known that plant secondary metabolites enable plants to reach their maximum antioxidant capacity. Consequently, several studies have shown a substantial correlation between the antioxidant capacity of plants and their plant secondary metabolites. It is sometimes necessary to conduct a number of studies in order to determine the in vitro antioxidant capability of plant resources [52]. The capability of a substance to scavenge free radicals indicates its antioxidant properties and ability to stop an oxidation chain from starting. The ABTS^•+^ and DPPH^•^ scavenging assays are frequently used to determine a chemical’s capacity to scavenge radicals [53]. The antioxidant capacity of *L. cariensis* essential oil was assessed using both scavenging assays. These are the most effective and widely used screening tests for radical elimination. They are employed to evaluate the capacity of a material to scavenge radicals. The concentration of *L. pinnate* essential oil needed to inhibit 50% of the DPPH radical was found to be 148.33 ± 2.48 μg/mL in another study, which indicates that the essential oil has a better antiradical activity [54]. *L. stoechas* essential oils were shown to have a significant RSA (IC_50_ = 221.43 μg/mL) in research involving alloxan-induced diabetic mice; nevertheless, this was less than that of the standard compound, ascorbic acid (IC_50_ = 87.57 μg/mL) [55]. Regarding IC_50_ values of 163.46 ± 5.66 µg/mL, the DPPH^•^ scavenging assay findings demonstrated that the antioxidant activity of essential oil from the Moroccan herbal plant *L. stoechas* was able to scavenge the DPPH free radicals [55]. Indeed, *L. multifida* essential oil scavenged DPPH radicals with concentrations of 15.23 μg/mL [56]. By IC_50_ values of 0.53 ± 0.01 mg/mL for *L. stoechas* and 0.284 ± 0.009 mg/mL for *L. dentata* in the DPPH^•^ scavenging test, compared to the standard antioxidants, *L. stoechas* and *L. dentata* essential oils had the highest antioxidant activity [57]. The essential oils of Algerian *L. stoechas* showed a very low antioxidant activity with an IC_50_ = 4.04 ± 0.047mg/mL for DPPH and 5.62 ± 0.08 mg/mL for ABTS, respectively [58]. *L. abrialis* and *L. stoechas* essential oils were extracted, and their DPPH activities were examined. The IC_50_ values demonstrated that *L. stoechas* essential oil was considerably better (IC_50_ = 12.94 μg/mL) than *L. abrialis* essential oil (IC_50_ = 34.71 μg/mL) and that ascorbic acid had a greater antioxidant capacity (1.62 μg/mL) than the essential oils of the two examined lavenders. The chemical components that make up extracted oils may have an impact on their antioxidant activity [59]. The essential oils of lavandin (Lavandula × intermedia ‘Grosso’) showed concentration-dependent antioxidant capacity in both the DPPH and ABTS tests [60]. The *L. angustifolia* essential oils ‘ IC_50_ values for DPPH and ABTS were 7.75 ± 0.10 and 18.71 ± 2.15 µg/mL, respectively, indicating their antioxidant activity [61]. Five varieties of *L. stoechas* cultivated in Thailand had their essential oils characterised. According to the DPPH and ABTS tests (IC_50_ of 67.65 and 89.26 mg/mL, respectively), the essential oil of *L. stoechas × viridis* “St. Brelade” had the best antioxidant capacity [62]. With values of 28.71 (IC_50_, µg/mL) and 8.72 (IC_50_, µg/mL), respectively, the ethyl acetate extract of *L. stoechas* demonstrated significant DPPH^•^ scavenging and ABTS^•+^ scavenging capabilities [10]. In a different study, it was demonstrated that all *L. species* methanol extracts show antioxidant activity, and the highest ability to scavenge free radicals was obtained for *L. viridis* and the lowest for *L. angustifoliae* [63]. When compared to the results of previous investigations, our *L. cariensis* essential oils data also have beneficial and efficacious reducing power (Fe^3+^, Cu^2+^, and FRAP) and antioxidant (ABTS^•+^ scavenging and DPPH^•^ scavenging activities) capabilities. Radical scavenging is crucial for reducing oxidative stress and protecting cells from damage associated with various diseases [64].

The antioxidant activity of *L. cariensis* essential oil can be better interpreted through comparison with other well-studied *Lavandula* species. In the present study, the DPPH^•^ scavenging activity of *L. cariensis* essential oil (IC_50_ = 231.0 μg/mL) was comparable to that reported for *L. stoechas* essential oil (IC_50_ = 221.43 μg/mL) in alloxan-induced diabetic mice studies [10], but lower than that reported for *L. multifida* essential oil (IC_50_ = 15.23 μg/mL) [65] and *L. angustifolia* essential oil (IC_50_ = 7.75 μg/mL) [11,13,66]. In contrast, the ABTS^•+^ scavenging activity of *L. cariensis* (IC_50_ = 7.45 μg/mL) was stronger than that reported for *L. angustifolia* (IC_50_ = 18.71 μg/mL) and several other lavender essential oils, indicating a considerable electron-donating capacity.

Enzyme inhibition is one of the most used and effective techniques in research on drug creation and manufacturing. Inhibiting certain metabolic enzymes can lessen the symptoms of several diseases, including obesity, diabetes, and problems associated with diabetes [67]. Hepatotoxicity and gastrointestinal issues are among the adverse effects of several synthetic inhibitors that have been reported. It is important to develop novel, naturally occurring inhibitors from natural chemicals that have no negative side effects [68]. The results of the enzyme inhibition analyses were examined and contrasted with the results from traditional inhibitors. It was found that *L. cariensis* essential oil efficiently inhibits AChE, α-amylase, and hCA II, which are connected to prevalent metabolic illnesses like AD and T2DM.

Nevertheless, the results of the earlier study did indicate that oregano’s capacity to inhibit cholinesterase can be influenced by the type of extraction method used. AChE inhibition was demonstrated to be more powerful when oregano leaves were utilised; water extracts demonstrated less inhibition. Regardless of very low concentrations, the oils of *O. ehrenbergii* and *O. syriacum* had an interesting inhibitory effect on AChE and BChE, two crucial enzymes in the pathophysiology of AD [69]. In a study of Lamiaceae family members, the AChE inhibitory potential of *L. stoechas* was found to be IC_50_ = 0.221 ± 0.01 mg/mL [70]. The active form of caspase-3 protein, which is recognised to be elevated in beta-amyloid-associated toxicity in an animal model, was also found to be reduced by linalool, one of the main constituents of *L. officinalis* essential oil [71]. *L. cariensis* may be a helpful source of antioxidants since it functions as a cholinesterase inhibitor. These results showed that *L. cariensis* oil was a strong and suitable AChE inhibitor when compared to tacrine.

Inhibition of α-amylase by natural products can effectively reduce blood glucose levels, especially for T2DM and postprandial glucose levels. *L. cariensis* essential oil demonstrated a more inhibitory effect than the common inhibitor acarbose (IC_50_: 22.800 mM) [72]. Several studies separated the soluble dietary fibres (SDF) and insoluble dietary fibres (IDF) from ripe *L. cariensis* oil using an in vitro digestion approach to see if they had an inhibiting impact on the enzyme α-amylase. It was demonstrated that both IDF and SDF had dose-dependent reductions in initial starch hydrolysis acceleration. Applying IDF at the same dose resulted in a stronger α-amylase inhibitory effect [73]. Inhibiting the activity of α-amylase, an enzyme that hydrolyses carbohydrates, is a potential treatment strategy for lowering blood glucose levels and managing T2DM. In fact, *L. multifida* essential oil showed an antidiabetic effect with IC_50_ values of 85.34 µg/mL against α-amylase [57]. With IC_50_ values of 3.00 ± 0.04 mg/mL, Moroccan *L. stoechas* essential oil demonstrated strong antidiabetic activity against α-amylase [56]. The essential oil’s IC_50_ for α-amylase inhibition was found to be 31.56 ± 0.46 μg/mL, whereas acarbose, the positive control, showed comparable efficacy with an IC_50_ of 35.48 ± 0.69 μg/mL. These findings imply that *L. pinnata* essential oil has a significant ability to inhibit α-amylase, an enzyme attached to the decomposition of carbohydrates [64]. The results of the investigation showed that *L. stoechas* essential oils were preventive towards oxidative stress and hyperglycemia in rats treated with alloxan [11]. In this study, we realised that *L. cariensis* essential oil inhibited the α-amylase enzyme, another diabetes regulator enzyme that can control and stabilise glucose levels. The inhibitory effects of the digestive enzymes, especially on α-amylase, may reduce blood glucose levels. Its suppression may also greatly enhance the management of hyperglycemia associated with diabetes. Medicinal herbs may be a useful treatment for diabetes and its complications, according to ongoing investigations conducted worldwide. Although α-amylase is a key enzyme involved in the initial digestion of dietary starch and is widely used as a target in antidiabetic screening studies, the inhibition of α-glycosidase also plays an important role in regulating postprandial glucose levels. Therefore, the antidiabetic activity reported in the present study should be interpreted as a preliminary evaluation based solely on α-amylase inhibition. Future investigations incorporating both α-amylase and α-glycosidase assays will provide a more comprehensive understanding of the potential antidiabetic effects of *L. cariensis* essential oil.

The antioxidant and enzyme-inhibitory activities observed for *L. cariensis* essential oil are generally consistent with previous reports on other *Lavandula* species; however, notable differences were observed in potency. For example, the ABTS^•+^ scavenging activity of *L. cariensis* essential oil (IC_50_ = 7.45 μg/mL) was comparable to or stronger than several previously reported Lavandula essential oils, whereas its DPPH^•^ scavenging activity was relatively moderate. Similarly, the AChE-inhibitory activity demonstrated by *L. cariensis* oil (IC_50_ = 14.22 μg/mL) suggests a promising cholinesterase-inhibition potential compared with related species. These differences may be attributed to variations in essential oil composition, particularly the high abundance of camphor and fenchone, as well as environmental, geographical, and genetic factors affecting metabolite production.

Regarding enzyme inhibition, direct comparisons are limited because most previous studies on lavender species have focused primarily on antioxidant, antimicrobial, and neuroprotective properties. Nevertheless, the AChE inhibitory activity observed for *L. cariensis* essential oil (IC_50_ = 14.22 μg/mL) suggests a noteworthy cholinesterase inhibitory potential. Likewise, the inhibition of hCA II (IC_50_ = 276.42 μg/mL) and α-amylase (IC_50_ = 475.63 μg/mL) provides additional evidence that *L. cariensis* may represent a valuable source of multifunctional bioactive compounds. Collectively, these comparisons demonstrate that *L. cariensis* exhibits biological activities within the range reported for other lavender species, while displaying a distinctive activity profile that may be attributed to its unique chemical composition. From a pharmacological perspective, the observed hCA II inhibitory activity is noteworthy because CA II plays a central role in aqueous humour secretion, and inhibition of this isoenzyme represents an established therapeutic strategy for reducing intraocular pressure in glaucoma patients [73]. Likewise, inhibition of AChE may enhance cholinergic neurotransmission by increasing synaptic acetylcholine levels, which forms the basis of currently approved symptomatic therapies for AD [74,75]. Furthermore, α-amylase inhibition is considered a recognised approach for delaying carbohydrate digestion and attenuating postprandial hyperglycemia, thereby contributing to glycemic control in T2DM [76].

Nevertheless, it should be emphasised that the present study evaluated the biological activities of the whole essential oil rather than isolated constituents. Therefore, a direct mechanistic link between individual compounds, such as camphor or fenchone, and the observed enzyme-inhibitory effects cannot be conclusively established. The current findings should be regarded as preliminary in vitro biochemical evidence supporting further studies involving bioactivity-guided fractionation, molecular interaction analyses, and in vivo validation.

Although lavender essential oils have been used for many years in traditional medicine, food products, cosmetics, and aromatherapy, their biological activities should be evaluated considering the available safety and pharmacokinetic data. Lavender essential oils are generally regarded as having low acute toxicity when used at appropriate concentrations, and several regulatory agencies have reported that their major constituents are safe within established exposure limits. In the present study, camphor and fenchone were identified as the predominant constituents of the essential oil. Both compounds are naturally occurring oxygenated monoterpenes that can be absorbed through oral, dermal, and inhalation routes, metabolised in the liver, and excreted primarily as conjugated metabolites. However, excessive exposure to camphor-containing products has been associated with neurotoxicity, particularly at high doses, whereas fenchone may cause mild gastrointestinal or neurological adverse effects when consumed in excessive amounts. Therefore, although the observed antioxidant and enzyme-inhibitory activities are biologically promising, the present findings should primarily be considered as preliminary in vitro observations. Further animal studies and clinical investigations evaluating bioavailability, metabolism, safety, and efficacy are required before any therapeutic applications can be considered.

The biological activities observed for *L. cariensis* essential oil may be associated, at least in part, with its major constituents, particularly camphor (39.73%) and fenchone (19.49%), which together account for approximately 59% of the total oil composition. Both compounds belong to the class of oxygenated monoterpenes, which are known to contribute to the biological properties of many Lavandula essential oils. Previous studies have demonstrated that camphor-rich lavender oils exhibit antioxidant and enzyme-modulating activities, partly through radical-scavenging mechanisms and interactions with biological targets [14,42]. Likewise, fenchone has been reported as an important bioactive constituent of several lavender species and may contribute to neuroprotective and enzyme inhibitory effects. The bicyclic oxygenated structures of camphor and fenchone confer moderate lipophilicity, which may facilitate interactions with hydrophobic regions of enzyme active sites. However, essential oils are highly complex mixtures, and their biological activities are generally attributed not only to the major constituents but also to synergistic and additive interactions among minor components [42,76]. Therefore, the antioxidant and enzyme-inhibitory activities observed for *L. cariensis* essential oil are likely the result of the combined action of its entire phytochemical profile rather than the effect of a single compound.

A recent study investigated the chemical composition and antioxidant potential of *Phlomoides rotata* essential oil using a metabolomic-guided approach combined with in vitro antioxidant assays. Fractionation of the essential oil by cryoprecipitation produced crystal and crystal-free fractions, allowing a more detailed characterisation of its constituents. GC–MS analysis identified 125 compounds, representing 84.41–94.86% of the total oil composition, of which 94 compounds were reported for the first time in *P. rotata*. The essential oil was found to be dominated by long-chain fatty acids (42.33–75.73%) and their esters (3.44–15.21%), with palmitic acid (14.49–63.13%), myristic acid, linoleic acid, oleic acid, and methyl palmitate being the major constituents. The authors suggested that these metabolites may contribute significantly to the observed antioxidant activity of the essential oil [77]. Unlike the fatty acid-rich profile reported for *P. rotata*, the essential oil of *L. cariensis* was characterised predominantly by oxygenated monoterpenes, particularly camphor and fenchone, suggesting that different classes of metabolites may underlie the antioxidant properties observed among aromatic plant species. Also, it was reported that the antioxidant activity of essential oils is closely associated with their ability to interfere with lipid peroxidation and maintain cellular redox balance [78]. Recent evidence indicates that, beyond direct radical scavenging, essential oil constituents may act through chain-breaking mechanisms and modulation of redox-sensitive pathways such as Nrf2/Keap1 [79,80] signalling. Phenolic compounds generally exhibit stronger radical-trapping capacity, whereas certain terpenes contribute through alternative mechanisms, including enhancement of radical termination and suppression of lipid oxidation [81]. The antioxidant activity of essential oils is mainly attributed to their ability to interrupt lipid peroxidation through radical-trapping mechanisms. Phenolic constituents donate hydrogen atoms to lipid peroxyl radicals, while certain terpenes contribute through alternative pathways. Additionally, modulation of redox-sensitive signalling pathways may enhance cellular antioxidant defences and oxidative stress resistance [75].

## 4. Materials and Methods

### 4.1. Chemicals

α-Tocopherol, Trolox, butylated hydroxyanisole (BHA), 1,1-diphenyl-2-picrylhydrazyl (DPPH), and butylated hydroxytoluene (BHT) were all bought from Sigma-Aldrich Chemie GmbH (Steinheim, Germany). The following chemical was bought from Sigma-Aldrich: 2,2′-azino-bis(3-ethylbenzthiazoline-6-sulfonic acid) (ABTS). Acetylcholinesterase (AChE) and α-amylase were obtained from Sigma. Acetylcholinesterase enzyme and α-amylase were commercially obtained—from *Electrophorus electricus* and horse serum, respectively—as specified by the manufacturer (Sigma). hCA II isoenzyme was purified from human erythrocytes using Sepharose-4B-L-tyrosine-sulfanilamide affinity chromatography. The corresponding details have now been added to the revised manuscript to ensure transparency and reproducibility of the experimental procedures.

### 4.2. Plant Material and Isolation of Essential Oil

The aerial parts of *Lavandula pendunculata* subsp. *cariensis* (syn. *L. cariensis*) was harvested in Aydın Province in June 2024 and has been identified by Prof. Dr. Tuncay Dirmenci, Balıkesir University. A voucher specimen has been deposited in the Herbarium Balikesir University, as TD 6050b. The harvested lavender was cleaned and dried in the shade. The *L. cariensis* essential oil was produced using steam distillation. Using steam as the stripping gas, this method separates the essential oils through many stages of continuous distillation. Direct steam exposure is given to the plant. The lavender was set on a grate above the steam distillation unit’s input. For around 120 min, the device received steam. The mixture of vapours is collected and condensed to create a liquid with two distinct coatings of oil and water. These coatings are composed of hydrophilic chemicals found in a hydrolysate or hydrosol and hydrophobic compounds detected in essential oils. Cohobating makes it possible to collect any polar compounds that remain in the water. The dried *L. cariensis* essential oil grid was placed over the steam inlet of the steam distillation device. The equipment received steam for about two hours. The mixture, which contained water vapour and volatile essential oils, was then collected in the gathering bottle after being condensed using a cooler. The *L. cariensis* essential oil obtained in the collecting container differentiated from the water phase and was subsequently removed due to the differing densities of water and oil.

### 4.3. Essential Oil Isolation and GC/MS and GC-FID Analyses

The extracted essential oil was dried over anhydrous CaCl_2_, and the essential oil was stored at 4 °C until the GC-MS/FID analyses. It yielded 1.52% of essential oil. A Thermo Scientific Trace GC 1310 (Thermo Fisher Scientific, Waltham, MA, USA) was supplied.connected to a Thermo TSQ 9610 MS (Thermo Fisher Scientific, Waltham, MA, USA) was supplied.device was used for the GC-MS analysis on a DB-5 capillary column (60 m, 0.25 μm, 0.25 μm film thickness) utilising helium as the carrier gas (0.8 mL/min). The temperature of the GC oven was supposed to increase to 280 °C at a rate of 4 °C per minute after being kept at 80 °C for ten minutes. It stayed at that temperature for five minutes. A split ratio of 1:20 was adopted. The temperature of the injector was set at 250 °C. The spectra were obtained at a mass eV of 70. The mass range was *m/z* 35–650. The GC-FID analysis was performed using a Thermo Scientific Trace GC 1310 apparatus (Thermo Fisher Scientific, Waltham, MA, USA) was supplied. The FID detector’s temperature was 280 °C. To accomplish the same elution sequence as GC-MS, parallel auto-injection was performed twice on the same column using the same operating parameters. The relative percentage of the separated compounds was measured using the FID chromatograms [82,83]. Alkanes were used as reference points in the Relative Retention Indices (RRIs) calculation. The chemicals were identified by comparing retention times and mass spectra using real samples, the NIST and Wiley spectra, and data from the literature [84,85,86,87].

### 4.4. Reducing Ability Assays

*L. cariensis* essential oil’s Fe^3+^ reduction potential was determined by applying the Fe^3+^(CN^−^)_6_ complex reduction procedure [88,89]. First, stock solutions were prepared for use in experimental studies. To this end, stock solutions with a concentration of 1 mg/mL were prepared for essential oil and standard solutions. For this assay, 2.5 mL of phosphate buffer (0.2 M, pH 6.6), 2.5 mL of 1% potassium ferricyanide [K_3_Fe(CN)_6_], and varying concentrations (10, 20, and 30 μg/mL) of *L. cariensis* essential oil were added to test tubes. The mixtures were thoroughly mixed and incubated at 50 °C for 25 min. Subsequently, 2.5 mL of 10% (*w*/*v*) trichloroacetic acid was introduced to terminate the reaction. Following phase separation, 2.5 mL of the supernatant was carefully collected and mixed with 0.5 mL of 0.1% FeCl_3_ solution and 2.5 mL of distilled water. The reducing capacity of *L. cariensis* essential oil was evaluated by measuring absorbance at 700 nm using a spectrophotometric method.

The Cu^2+^ reducing capacity of *L. cariensis* essential oil was determined by measuring its absorbance according to procedures carried out according to the method of Apak et al. [90]. This was accomplished by transferring 0.25 mL of CuCl_2_ reagent mixture (10 mM), 0.25 mL of ethanolic neocuproine solution (7.5 × 10^−3^ M), and 250 μL of ammonium acetate buffer reagent (1.0 M) into test tubes comprising *L. cariensis* essential oil at different concentrations (10, 20, and 30 μg/mL). Following 30 min of incubation, the absorbance values were obtained at 450 nm after the total volume was increased to 2 mL with distilled water.

The potential of *L. cariensis* essential oil to be reduced by the Fe^3+^-2,4,6-tris(2-pyridyl)-S-triazine (TPTZ) combination was also investigated according to a previous work [91]. The ferric reducing antioxidant power (FRAP) assay was conducted using a freshly prepared reagent consisting of TPTZ (10 mM in 40 mM HCl), FeCl_3_ (20 mM), and acetate buffer (0.3 M, pH 3.6). The reagent was mixed with varying concentrations (10, 20, and 30 μg/mL) of *L. cariensis* oil and incubated at 37 °C for 25 min. Absorbance was measured at 593 nm using a UVmini-1240 UV-VIS spectrophotometer (Shimadzu Corporation, Kyoto, Japan), and the increase in absorbance was interpreted as an indicator of reducing capacity. All measurements were performed in triplicate, and results were expressed as mean values.

### 4.5. Radical Scavenging Activities

*L. cariensis* essential oils’ capacity to scavenge radicals was evaluated utilising the Blois method and the DPPH radicals [92]. The radical scavenging activity of *L. cariensis* essential oil was evaluated using the DPPH assay. Briefly, a 0.1 mM DPPH^•^ solution prepared in ethanol was used, and its initial absorbance was adjusted to 1.00 ± 0.200. Subsequently, 3 mL of the DPPH working solution was mixed with 0.5 mL of *L. cariensis* essential oil extract at varying concentrations (10, 20, and 30 μg/mL). The reaction mixtures were thoroughly mixed and incubated in the dark for 30 min at room temperature. The decrease in absorbance, corresponding to the scavenging of DPPH radicals, was monitored at 517 nm using a spectrophotometer. All measurements were performed in triplicate [93]. According to Gulcin’s methodology [94].

Also, the radical-scavenging capacity of *L. cariensis* essential oil was proven against ABTS radicals. An aqueous solution of ABTS (7.0 mM) was initially oxidised with K_2_S_2_O_8_ (2.5 mM) to form the radical cation ABTS^•+^. Before being used, the ABTS^•+^ solution was diluted with a phosphate buffer (0.1 M, pH 7.4) to adjust the absorbance value of the control solution to 0.750 ± 0.025 at 734 nm. Next, three millilitres of *L. cariensis* essential oil were mixed with different amounts (10, 20, and 30 μg/mL) of ABTS^•+^ solution. The absorbance of remaining ABTS^•+^ was recorded after half an hour at 734 nm [95].

The radical scavenging potential (RSC) of *L. cariensis* essential oil was calculated applying the following formula: RSC (%) = (1 − A_c_/A_s_) × 100, where A_c_ and A_s_ are the absorbance values of the control and sample, respectively. Additionally, IC_50_ was determined using the graphs and provided in µg/mL [96].

### 4.6. Acetylcholinesterase Inhibition Assay

The inhibitory capacity of *L. cariensis* essential oil toward the cholinergic AChE enzyme was determined by applying Ellman’s method [97]. *L. cariensis* essential oil was evaluated on AChE from *Electrophorus electricus* [98]. Acetylthiocholine iodide (AChI) and 5,5′-dithio-bis-(2-nitrobenzoic acid) (DTNB) were used as substrates. To this end, 50 μL of *L. cariensis* essential oil at different concentrations (10, 20, and 30 μg/mL), 50 μL of AChE, and 1 mL of Tris/HCl buffer (1.0 M, pH 8.0) were all added to a test tube. Following 15 min of incubation at 25 °C, the material was placed into 50 μL of DTNB solution (0.5 mM). 50 μL of AChI solution (10 mM) was poured to start the procedure, and the absorbances of samples were measured at 412 nm [99].

### 4.7. α-Amylase Inhibition Assay

The α-amylase inhibition capacity of *L. cariensis* oils was evaluated using starch as a substrate according to Xiao’s approach [100]. First, 1 g of starch was dissolved in 50 mL NaOH solution (0.4 M). The mixture was then heated to 80 °C for 20 min. After cooling, the pH and volume were adjusted to 6.9 and 100 mL, respectively, using distilled water. Next, 35 µL of the starch solution, 5 µL of *L. cariensis* essential oil, and 35 µL of phosphate buffer (pH 6.9) were mixed. After adding 20 µL of α-amylase solution, the mixture was incubated for 20 min at 37 °C. After 50 µL of 0.1 M HCl was applied to complete the reaction, absorbance was measured at 580 nm.

### 4.8. hCA II Inhibition Assay

As mentioned previously, Sepharose-4B-L-Tyrosine sulphanilamide affinity chromatography and laboratory-discarded human blood samples were used to separate and purify CA II isoenzymes [101]. Protein concentrations were measured at 595 nm using the Bradford method after the enzymes were purified [102]. An esterase activity experiment was conducted at 348 nm using a spectrophotometer (Shimadzu, UVmini-1240 UV-VIS, (Shimadzu Corporation, Kyoto, Japan) using the methodology of Verpoorte et al. [103]. As a reference standard, acetazolamide (AZA) was utilised.

### 4.9. Determination of IC_50_ Value

The inhibitory effects of *L. cariensis* essential oil were assessed using IC_50_ values. IC_50_ values were calculated from graphs displaying the enzyme activity as an outcome of rising *L. cariensis* essential oil concentration [104].

### 4.10. Statistical Analysis

All measurements were performed in triplicate, and the data are expressed as mean ± standard deviation (SD). Statistical analysis was conducted using one-way analysis of variance (ANOVA) followed by Tukey’s post hoc test. Differences were considered statistically significant at *p* < 0.05.

## 5. Conclusions

*L. cariensis* essential oil could serve as a promising natural source for developing antioxidant, neuroprotective, hypoglycemic, and antiglaucoma agents. Since it has a good taste and advantages in health, the essential oil demonstrated enzyme-inhibitory activities against targets associated with AD, diabetes, and glaucoma. Several in vitro bioanalytical tests were used to assess the antioxidant qualities of *L. cariensis* essential oil as well as its inhibitory efficacy on enzymes: AChE, α-amylase, and CA II, which are linked to diabetes mellitus, AD, and glaucoma, respectively. It was also possible to identify any putative active components in *L. cariensis* essential oil. Furthermore, *L. cariensis* essential oil’s GC-FID study revealed that camphor made up 39.73% of the total essential oil present. Additionally, *L. cariensis* essential oil contains a lot of naturally occurring plant secondary metabolites, the most prevalent of which are camphor, fenchone, exobornyl acetate, camphene, and eucalyptol. According to the findings, *L. cariensis* essential oil can be a valuable and potential source of biomolecules that are crucial to biological function. Also, this study’s in vitro inhibition suggests promising prospects.

This study has several limitations that should be considered when interpreting the findings. First, the biological activities of *L. cariensis* essential oil were evaluated exclusively through in vitro antioxidants and enzyme inhibition assays. Therefore, the observed effects cannot be directly extrapolated to in vivo efficacy or clinical applications. Second, the study focused on the overall essential oil composition rather than the individual contribution of specific constituents. Although camphor and fenchone were identified as the major components, their precise roles and possible synergistic or antagonistic interactions with minor constituents were not investigated. In addition, factors such as geographical origin, seasonal variation, harvesting stage, and environmental conditions may influence essential oil composition and consequently its biological activity. Furthermore, the pharmacokinetic properties, bioavailability, metabolism, and toxicity of the essential oil were not evaluated. Future studies involving bioactivity-guided fractionation, mechanistic investigations, toxicity assessments, and in vivo models are required to confirm and extend the present findings.

## Figures and Tables

**Table 1:** Chemical composition of Lavandula (*L. cariensis*) essential oil was recorded by utilising GC-MS.

RRI	Essential Oils	Formula	Contents%
933	Thujene	C_10_H_16_	0.68
942	α-Pinene	C_10_H_16_	2.71
953	Camphene	C_10_H_16_	5.49
990	β-Phellandrene	C_10_H_16_	0.15
1004	*p*-Cymene	C_10_H_14_	1.19
1013	Eucalyptol	C_10_H_18_O	4.11
1044	Linalool Oxide Trans	C_10_H_18_O_2_	1.19
1064	Fenchone	C_10_H_16_O	19.49
1069	Linalool	C_10_H_18_O	1.61
1087	D-Fenchyl alcohol	C_10_H_18_O	0.52
1098	Campholaldehyde	C_10_H_16_O	0.43
1114	*cis*-Sabinol	C_10_H_16_O	0.30
1121	Camphor	C_10_H_16_O	39.73
1141	1-Borneol	C_10_H_18_O	1.64
1158	*p*-cymene-8-ol	C_10_H_14_O	1.18
1164	α-Terpıneol	C_10_H_18_O	0.21
1171	Myrtenol	C_10_H_16_O	0.42
1185	Berbenone	C_10_H_14_O	1.68
1191	*trans*-Carveol	C_10_H_16_O	0.26
1194	Fenchyl acetate	C_12_H_20_O_2_	0.28
1220	Carvone	C_10_H_14_O	0.52
1228	Linalyl acetate	C_12_H_20_O_2_	1.44
1266	Exobornyl acetate	C_12_H_20_O_2_	6.81
1279	Thymol	C_10_H_14_O	0.27
1481	Valencene	C_15_H_24_	1.35
1499	2-Allyl-5-t-butylhydroquinone	C_13_H_18_O_2_	0.88
1536	Calacorene	C_13_H_16_	0.22
1580	Spathulenol	C_15_H_24_O	0.60
1762	Muscalure	C_23_H_46_	0.40
Total			96.44

**Table 2:** Fe^3+^, Cu^2+^, and Fe^3+^-TPTZ reducing ability of *L. cariensis* essential oil and standards at 30 μg/mL concentration (BHA: butylated hydroxyanisole, BHT: butylated hydroxytoluene).

Antioxidants	Fe^3+^ Reducing *		Cu^2+^ Reducing *		Fe^3+^-TPTZ Reducing *	
	λ_700_	r^2^	λ_450_	r^2^	λ_593_	r^2^
BHA	2.292 ± 0.012 ^c^	0.9993	2.418 ± 0.018 ^c^	0.9887	1.172 ± 0.014 ^c^	0.9605
BHT	2.136 ± 0.090 ^c^	0.9957	1.953 ± 0.045 ^b^	0.9998	0.690 ± 0.008 ^a^	0.9645
Trolox	1.514 ± 0.066 ^b^	0.9963	1.800 ± 0.096 ^b^	0.9974	1.180 ± 0.032 ^c^	0.9732
α-Tocopherol	0.862 ± 0.038 ^a^	0.9996	0.851 ± 0.046 ^a^	0.9994	0.918 ± 0.011 ^b^	0.9904
Ascorbic acid	2.298 ± 0.086 ^c^	0.9659	0.983 ± 0.048 ^a^	0.9822	1.257 ± 0.024 ^c^	0.9869
*L. cariensis* oil	2.397 ± 0.093 ^c^	0.9600	2.073 ± 0.105 ^b^	0.9995	1.318 ± 0.021 ^c^	0.9873

* All data are the averages of three parallel observations and are displayed as mean ± SD (*n* = 3). Values with different superscript letters differ significantly at *p* < 0.05.

**Table 3:** IC_50_ values (μg/mL) of the *L. cariensis* essential oil and standards for the scavenging of DPPH^•^ and ABTS^•+^ radicals.

Antioxidants	DPPH^•^ Scavenging		ABTS^•+^ Scavenging	
	_IC50_	r^2^	IC_50_	r^2^
BHA	6.86±0.006 ^a^	0.9762	6.36±0.011 ^a^	0.9450
BHT	49.50±0.022 ^b^	0.9155	12.60±0.032 ^b^	0.8668
Trolox	6.03±0.009 ^a^	0.5964	16.50±0.053 ^c^	0.9926
α-Tocopherol	7.70±0.011 ^a^	0.5211	18.73±0.107 ^c^	0.9082
Ascorbic acid	5.82±0.005 ^a^	0.5211	11.75±0.022 ^b^	0.9082
*L. cariensis* oil	231.00±0.094 ^c^	0.8669	7.45±0.013 ^a^	0.8820

Values with different superscript letters differ significantly at *p* < 0.05.

**Table 4:** The IC_50_ values (µg/mL) of *L. cariensis* essential oil versus α-amylase, acetylcholinesterase, and carbonic anhydrase II enzymes.

Enzymes	*L. cariensis* Oil		Standard Inhibitors	
	IC_50_	r^2^	IC_50_	r^2^
α-Amylase ^1^	475.63	0.9002	7.54	0.9074
Acetylcholinesterase ^2^	14.22	0.9552	8.82	0.9836
Carbonic anhydrase II ^3^	276.42	0.9055	9.96	0.9930

^1^ The enzymes α-glycosidase and α-amylase were positively controlled by acarbose. ^2^ Tacrine was used to evaluate the acetylcholinesterase enzyme. ^3^ Acetazolamide (AZA) was a positive control for the isoenzyme carbonic anhydrase II.

## Data Availability

The original contributions presented in this study are included in the article. Further inquiries can be directed to the corresponding authors.
